# Antimicrobial resistance profile of *Aeromonas* species isolated from Libya

**DOI:** 10.3402/ljm.v8i0.21320

**Published:** 2013-05-24

**Authors:** Khalifa Sifaw Ghenghesh, Hanan El-Mohammady, Samuel Y. Levin, Abdulazziz Zorgani, Khaled Tawil

**Affiliations:** 1 Faculty of Medicine, University of Tripoli, Tripoli, Libya. Email: ghenghesh_micro@yahoo.com; 2 US Naval Medical Research Unit-3, Cairo, Egypt; 3 Faculty of Medicine, University of Tripoli, Tripoli, Libya

*Aeromonas* species are Gram-negative, oxidase-positive rods in the family *Aeromonadaceae*. These organisms have been isolated from untreated drinking water and different types of foods particularly seafood and frozen chicken. *Aeromonas* species are recognized as agents of serious infections in immunocompromised individuals including septicemia in patients with hepatic cirrhosis with a rapidly fatal outcome (1). Significant association of *Aeromonas* species with diarrhoea, particularly in children, and soft tissue infections following water-related injuries were reported from several developing countries (2).

Most diarrheal cases due to aeromonads are self-limiting and treated by fluid and salts replacement. However, antimicrobial therapy should start for patients who are severely ill and for those with risk factors for extraintestinal spread of infection. There are few reports on the susceptibility of *Aeromonas* species to antimicrobial agents from Libya and other countries in North Africa and the Middle East. The present investigation was carried out to determine the antimicrobial susceptibility profile of *Aeromonas* species isolated from different sources in Libya.

Included in the study were 70 *Aeromonas* isolates from diarrheic children (*n*=22), non-diarrheic children (*n*=11), chicken carcases (*n*=18) and untreated drinking water (*n*=19). *Aeromonas* species were isolated from different sources using standard bacteriological procedures and identified to genus level (i.e. *Aeromonas* species) using API 20E and API 20NE as recommended by the manufacturer (bioMerieux, France). Susceptibility of *Aeromonas* isolates to antimicrobial agents was determined by the disc diffusion method recommended by the Clinical Laboratory Standards Institute (3).

In previous studies, fluoroquinolones, 3rd generation cephalosporins and aminoglycosides showed excellent activity against *Aeromonas* species isolated from clinical sources and foods (4, 5). Similar results obtained in the present investigation in which all *Aeromonas* isolates examined were susceptible to ciprofloxacin, ceftriaxone and gentamicin. Table 1 shows antimicrobial susceptibility profile of *Aeromonas* species isolated from Libya. We observed a significantly higher resistant rate to tetracycline among aeromonads from chicken carcases (33%, 6/18) compared with aeromonads from water (0.0%, 0/19) (*P*< 0.006, OR = Undefined), but not with aeromonads from diarrheic and non-diarrheic children (*P*> 0.05). Tetracyclines are common additives in feed for poultry, which may have contributed to the observed high rate of resistance to tetracycline among our *Aeromonas* isolates from chicken carcases. Resistance associated with antimicrobial growth promotants has been known for decades (6).

**Table 1:** Antimicrobial susceptibility profile of *Aeromonas* species isolated from Libya

	%														
	
Antimicrobial agent	Diarrheic children (*n*=22)			Non-diarrheic children (*n*=11)			Chicken (*n*=18)			Water (*n*=19)			Total (*n*=70)		
					
	S	I	R	S	I	R	S	I	R	S	I	R	S	I	R
Amoxicillin–clavulanic acid	40.9	45.5	13.6	54.5	36.4	9.1	27.8	72.2	0	7	31.6	31.6	38.6	47.1	14.3
Ceftriaxone	100	0.0	0.0	90.9	9.1	0.0	100	0.0	0.0	100	0.0	0.0	98.6	1.4	0.0
Ciprofloxacin	100	0.0	0.0	100	0.0	0.0	100	0.0	0.0	100	0.0	0.0	100	0.0	0.0
Gentamicin	100	0.0	0.0	100	0.0	0.0	100	0.0	0.0	100	0.0	0.0	100	0.0	0.0
Tetracycline	77.3	4.5	18.2	90.9	0.0	9.1	61.1	5.6	33.3	100	0.0	0.0	81.4	2.9	15.7
Trimethoprim–sulfamethoxazole	86.4	0.0	13.6	100	0.0	0.0	100	0.0	0.0	100	0.0	0.0	95.7	0.0	4.3

S = susceptible, I = intermediate susceptible, R = resistant.

Previous studies from the region reported 100% *Aeromonas*-resistance rates to ampicillin and other penicillins (7, 8). Most *Aeromonas* isolates are intrinsic or chromosomally mediated resistant against ampicillin (9). Of the total *Aeromonas* isolates examined in the present study, 14.3% were resistant and 47.1% were intermediate susceptible to amoxicillin–clavulanic combination (i.e. <40% susceptible). Therefore, in cases of water-related wound infections not responding to treatment with ampicillin, amoxicillin or amoxicillin–clavulanic combination, physicians should suspect *Aeromonas* as the causative agent. Although, ciprofloxacin and 3rd generation cephalosporins are excellent antimicrobials in the treatment of *Aeromonas*-associated infections, trimethoprim-sulfamethoxazole appears a viable option for the treatment of such infections.

*Khalifa Sifaw Ghenghesh* Faculty of Medicine University of Tripoli Tripoli, Libya Email: ghenghesh_micro@yahoo.com *Hanan El-Mohammady* US Naval Medical Research Unit-3 Cairo, Egypt *Samuel Y. Levin* US Naval Medical Research Unit-3 Cairo, Egypt *Abdulazziz Zorgani* Faculty of Medicine University of Tripoli Tripoli, Libya *Khaled Tawil* Faculty of Medicine University of Tripoli Tripoli, Libya
